# The Impact of Tween 20 on the Physical Properties and Structure of Agar Gel

**DOI:** 10.3390/gels11030159

**Published:** 2025-02-23

**Authors:** Ewa Jakubczyk, Anna Kamińska-Dwórznicka, Anna Kot

**Affiliations:** 1 Department of Food Engineering and Process Management, Institute of Food Sciences, Warsaw University of Life Sciences, 02-776 Warsaw, Poland; anna_kaminska1@sggw.edu.pl; 2 Department of Functional and Organic Food, Institute of Human Nutrition Sciences, Warsaw University of Life Sciences, 02-776 Warsaw, Poland; anna_kot1@sggw.edu.pl

**Keywords:** porous gels, agar, aeration, Tween 20, texture, rheology, structure

## Abstract

This study aimed to evaluate the effect of different concentrations of Tween 20 on various physical properties of agar gel as a model material. The effects of other sources of agar-agar powder on the gel properties were also evaluated. The pure gels were prepared with agar powders obtained from two suppliers. Also, agar gels with Tween 20 in the 0.10 to 0.70% range were produced. The measurement of density, water activity, maximal force at fracture and gelling temperature, and the agar gels’ rheological properties, showed that the gels prepared with different agar powders had similar properties. The syneresis and stability indexes, gas hold-up, mechanical and acoustic attributes, and structure of foamed gels with Tween 20 were measured. The addition of Tween 20 in amounts ranging from 0.10 to 0.35% contributed to a gradual decrease in the stability and mechanical parameters of the gels. Using a concentration of 0.7%, Tween was able to obtain foamed gels with a uniform structure and small pore size, but low hardness and gumminess. Application of a lower concentration of Tween of 0.1% produced more rigid gels with limited gel syneresis. Adding Tween 20 at the appropriate level can be a factor in obtaining gels with a tailored structure and texture.

## 1. Introduction

Gels and gelled materials can find many applications in different fields, such as food, cosmetics, biomedicine production, templates for nanomaterials and many others [1]. Hydrocolloids are ingredients that are commonly used to prepare food gels. They have strong hydrophilic properties and can form a gel matrix by being in contact with water. The presence of hydrophilic colloids leads to the production of colloidal dispersions, which are intermediate forms between true solutions and suspensions. The main application of hydrocolloids relies on their ability to modify the rheological properties, texture, structure and sensory attributes of fabricated foods [2]. Plant-origin hydrocolloids are particularly popular because they are used to fabricate food for vegetarians. Among the wide range of these groups of hydrocolloids, agar-agar deserves attention [3]. Agar is a natural gelatinous polysaccharide, produced from many red algal species, that can form thermo-reversible hydrogels by heating and cooling [4]. Agar gels are also rigid and stable at various temperatures. The rheological and mechanical behaviour of gels during mastication can be linked with their functionality [5]. The structure and textural properties of agar gels make them suitable for preparing foods for patients with swallowing problems [6]. Gels at low agar concentrations are characterised by high elastic moduli values, which are especially crucial in the food design process [7].

The rheological behaviour of hydrocolloids is of great importance when modifying the textural properties of foods [8]. Using gelling agents allows the production of new and attractive forms of food that are acceptable to consumers [3]. Gels’ mechanical and rheological properties can be determined using different techniques based on small or large strain deformation. Dynamic rheology at small deformations can effectively describe the viscoelastic properties of gels and changes in their properties during the gelation process. The mechanical attributes obtained during compression tests and texture profile analysis (TPA) can be linked with many sensory descriptors perceived by consumers [9]. Acoustic techniques are an additional tool in assessing food texture. The acoustic signal generated during the deformation of foods can contain information about some textural features of materials and their structure. The acoustic emission method evaluates texture properties like crispness, crunchiness and hardness [10]. However, a strong correlation between structural changes and intensity of acoustic emission has also been observed [11,12,13].

Control of the gelling mechanisms and properties of gels is essential when designing materials for various applications. Application of a specific stimulus, e.g., ultrasound, light, pH change, or addition of ions, is a method of manipulating gelation. However, these techniques can be ineffective in the production of bulk gels. Surfactants are commonly used to fabricate different materials, but their impact on gelation is less known [14]. Enhanced fibre tip branching and an increase in the G′ of molecular gels were observed by adding the non-ionic surfactant Tween 80 [15]. Polyoxyethylene sorbitol esters 20 and 80 (commercially known as Tween) were used as emulsifiers to prepare gelled emulsions [16]. Tween surfactants are frequently used in the food industry and in pharmaceutical and cosmetic preparations. Polyoxyethylene (20) sorbitan monolaurate (Tween 20) is a non-ionic surfactant that characterises water solubility better than other Tweens. This surfactant has a high hydrophilic–lipophilic balance, which makes it suitable for oil-in-water emulsions [17]. Tweens can also be added as stabilising and foaming agents. However, foams generated by other Tweens are unstable. Tween 20 can stabilise foams by a monomolecular layer controlled by the Gibbs–Marangoni mechanism [18]. The foaming power and foamability of solutions with Tween 20 (also known as Polysorbate 20) have been analysed. An increase in surfactant concentration from 0.01 to 1% caused the foam volume to increase, but the foam’s stability was best at the lowest concentration of Polysorbate 20. Tween 20 also had better emulsifying power than Tween 60 and Tween 80, due to higher adsorption ability at the interface phase. This led to better stability of the system [19]. Adding surface-active substances (Tweens, mono- and di-glycerides, proteins) can improve foam stability and foaming capacity [20]. The concentration of foaming agents can affect the structure and texture of foams [21].

This study aimed to evaluate the effect of different concentrations of Tween 20 (Polysorbate 20, Polyoxyethylene sorbitan monolaurate 20) on selected physical properties of foamed agar gel as a model material. The effects of different sources of agar-agar powder on the gel properties were also evaluated.

## 2. Results and Discussion

### 2.1. Characteristic of Selected Properties of Agar Gel A and B

Agar can be extracted from different red algal groups (*Gracilaria*, *Gelidium*, *Gelidiella Pterocladia*) [22]. Many studies have shown that agar yield and its properties depend on species [23], environmental parameters [24] and methods of extraction [22,25]. In the current work, due to the availability of agar-agar powder from two suppliers, its fundamental physical properties were checked.

The water activity of the two agar gels was similar, and ranged from 0.984 to 0.988 (Table 1). The high water activity value was related to the composition of gels and the low amount of added dry matter (2% of agar powder). The density and the syneresis index did not differ significantly for samples A and B (Table 1). Aranda-Lara et al. [26] noted that the density of 2.33% agar gel was 0.999 g/cm^3^, which was a lower value than observed for gels A and B. Pure agar gels with a higher density of 1.29 g/cm^3^ were also prepared as a material for texture experiments [11,27]. Different manufacturers supplied the agar powder used in these studies. Differences in the physical parameters of gels may also result from the gel preparation method and the density measurement method.

The mechanical resistance expressed by maximal force recorded at gel fracture was similar for the two analysed agar gels (Table 1). However, Marinho-Soriano and Bourret [22] observed that agar strength varied from 101 to 543 g/cm^2^ for powder obtained from different species of algae.

The rheological behaviour of a gel is essential in fabricating the texture and structure of new products [7]. Also, sol–gel and gel–sol–gel transitions affect the rheological properties of the material. The gelation and liquefaction temperatures can be determined based on the changes in the rheological behaviour of the samples during this process [28,29].

**Figure 1:**
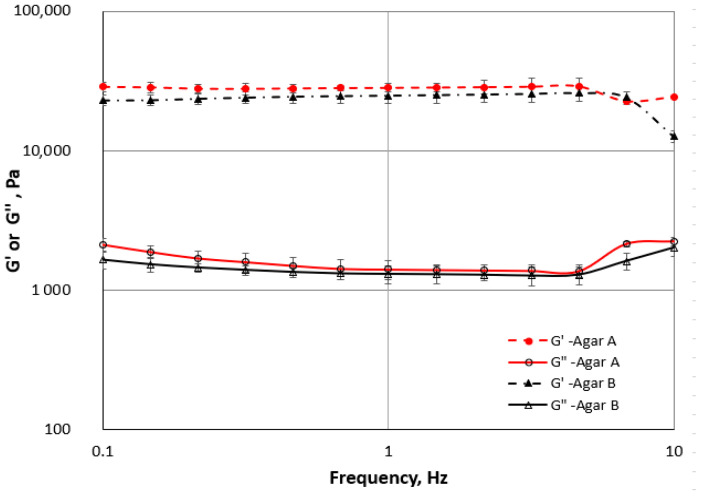
Frequency sweep curves for agar gels A and B.

Figure 1 presents the frequency sweeps of agar gels A and B. The values of G′ significantly exceeded the G″ values in the entire range of investigated frequencies. This is typical for a gel-like spectrum [30]. Bertrasa et al. [31] also observed significant dominance of the storage modulus G′ over the loss modulus G″ of agar hydrogels throughout the whole frequency range. Figure 1 shows that the values of G′ and G″ were independent of frequency changes over a wide range of this variable. It indicates the pure elastic behaviour of the gels [30,32]. The course of G′ and G″ changes with increasing frequency was similar for agar gels A and B.

**Figure 2:**
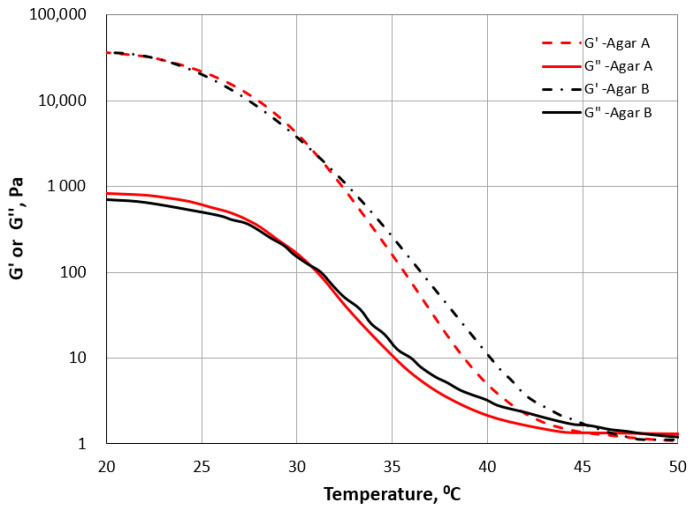
Temperature sweep curves for agar gels A and B.

The rheological behaviour of the agar samples was analysed based on the cooling of the material from 50 to 20 °C (Figure 2). The decrease in temperature caused an increase in G′ and G″. The change in the course of the G′ and G″ curves during cooling (intersection of the curves) indicates a phase transition. The gelling temperature was 45.4 ± 0.3 and 45.1 ± 0.1 °C for the agar gel A and B samples. Other studies have shown that the gelling temperature of 1.5–2% agar gels varied from 33 °C [33] to 45.3 °C [28]. The mean gelling temperature for agar extracted from different red algae was about 40 °C. However, the temperature varied between seasons from 34 °C (summer) to 46 °C (autumn) for agar extracted from *Gracilaria bursa-pastoris* [22].

### 2.2. Comparision of Agar Gels A and B with Tween 20

**Figure 3:**
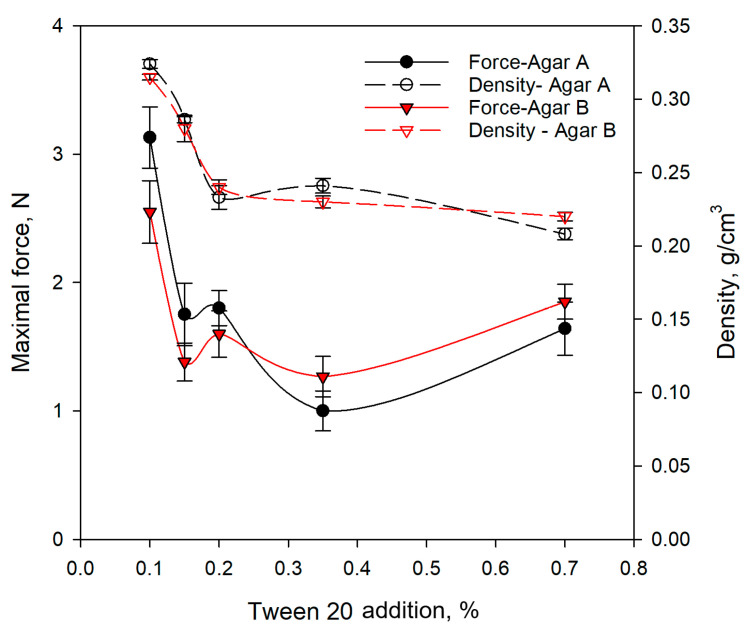
Maximal force of compression test for agar A and agar B gels with different additions of Tween 20.

Based on the results obtained for pure agar gel, which showed similar behaviour of both types of gels A and B, a gelled material with the addition of Tween 20 was prepared, and the density and strength of the material were determined (Figure 3). The concentration range of Tween 20 was selected based on preliminary research. Adding Tween 20 at a concentration above 0.75% resulted in a very soft and unstable material. The obtained product did not form a gel.

The addition of Tween 20 caused a reduction in the density of pure agar gels 4 ÷ 7 times, depending on the surfactant concentration (Table 1, Figure 3). An increase in the addition of Tween 20 from 0.1 to 0.20 caused a decrease in the density of the foamed material for both types of gels. The same tendency was observed for albumin, where an increase in the concentration of the foaming agent caused a reduction in gel density [27]. Further incorporation of the foaming ingredient Tween 20 at a concentration from 0.20 to 0.70% led to a slight density fluctuation from 0.240 to 0.210 g/cm^−3^ (Figure 3).

The apparent density of aerated agar–fructose gels decreased with an increase in the whipping time to 5 min. More extended aeration led to a slight increase in the density of gels, which was caused by the aggregation of tiny bubbles and fractures of bigger ones [11]. A decreased foam density with increased albumin concentration was also observed for aerated plant extract. The optimum albumin concentration of 12.2% enabled the obtention of the lowest foam density of 0.25 g/cm^3^; a higher addition of foaming ingredient caused equalisation of or a decrease in foam density [34]. A similar course of changes was observed for maximal force (at fracture of gel). However, a significant decrease in force was observed at the increase in Tween 20 concentration from 0.1 to 0.15–0.20%. The higher incorporation of Tween 20 caused some fluctuation in maximal force (Figure 3). The incorporation of bubbles into the gel matrix led to a decrease in hardness. A decrease in the density of aerated agar gels causes a reduction in failure force by 5 times [27]. The maximal force obtained for pure agar gel was considerably higher (24 N) than for gels with Tween 20 (1 ÷ 4 N). This may indicate that the aerated samples were softer than the pure agar gels. Aerated gelatine gels also had lower mechanical strength and a more brittle structure than non-aerated samples, due to the lower matrix content in gels [35]. The addition of surfactant to gels caused a significant reduction in compression force, due to the presence of voids and consolidation of the material during compression. The effect of lower mechanical strength was also linked with the introduction of bubbles. The gel structure bonded the water and surrounding gas bubbles, which led to weakening of the gel matrix. However, the structure of the foamed gel, with different concentrations of Tween 20, determined the material’s texture.

### 2.3. Characteristics of Selected Physical Properties of Agar Gel A with Tween 20

Instrumental Texture Analysis parameters have shown a strong correlation with sensory attributes of texture, and can provide more information than other gel strength measurements [36]. The TPA results of the agar A gels showed that the hardness of materials decreased with increasing concentration of Tween 20 (Table 2). The highest hardness, gumminess and springiness were obtained for agar gel without Tween 20. The incorporation of bubbles reduced the mechanical strength. However, the changes in the hardness with an increase in Tween 20 addition in the range from 0.15 to 0.7% were insignificant. The hardness and gumminess of the gels slightly decreased with an increase in the addition of foaming ingredients. Gumminess characterises the energy necessary to disintegrate and swallow the food pieces [37]. The springiness of gels with the addition of Tween 20 was at a constant level up to a concentration of 0.20%. An increase in foaming agent caused fluctuations in this parameter. The lowest springiness was observed for the gel with the highest addition of Tween 20. A low value of this parameter indicates that the gel breaks up into many small pieces. Less springy gels, which include carrageenan and agar gels, break up more quickly when chewed in the mouth. Cohesiveness describes the toughness of the material and its ability to be destroyed [37]. The cohesiveness of gels with Tween 20 was almost constant up to a concentration of surfactant of 0.35% (Table 2). A higher incorporation of foaming agent caused a decrease in this parameter. The bubbles incorporated during the stirring of sol with foaming agent Tween 20 caused weakening of the gels, with a reduction in their hardness, gumminess and cohesiveness. A similar effect was observed for gels with a chokeberry gel with albumin [27].

The acoustic properties can be related to the composition and structure of deformed materials. The fracture of materials recorded during the compression of samples may contain acoustic information about the samples’ internal structure and texture [38]. Table 3 shows acoustic descriptors obtained for the gels with the addition of Tween 20. Due to overlap between the signal from the materials and the acoustic background, data for the control gel were not included. The number of acoustic events decreased with the increased addition of foaming agents from 0.10 to 0.20%. The highest total energy and number of AE events generated during the deformation of samples was observed for samples with the highest addition of Tween 20 (0.7%). A low concentration (0.1%) of Tween 20 caused the generation of shorter acoustic impulses compared to gel with a higher addition of surfactant. The acoustic signal emitted during the deformation of aerated gels can be linked with breaks in the hydrocolloid matrix, cracking of bonds and the internal structures of the material [11,39].

The stability of gels after production and storage is a crucial quality feature. The syneresis values and TSI indicated that increasing the amount of foaming agent caused the gel structure to have lower stability, with higher solvent leakage. High TSI values indicate undesirable phenomena, e.g., flocculation and coalescence in gels [40,41]. The higher value of TSI obtained for the agar gel indicates lower stability of the material’s structure than observed for the aerated samples during storage. However, the solvent leakage from the agar gel after centrifugation was lower than that of the aerated samples. The most unfavourable changes were observed at 0.35% Tween 20 (Table 4). An increase in the addition of this ingredient led to a rise in the gel’s stability. The results indicate that too-high and too-low surfactant concentrations adversely affect water retention in the gel matrix.

The addition of Tween 20 affected the gas hold-up of gels (Table 4). An increase in foaming agent addition led to an increase in this parameter. This means that a greater gas fraction was incorporated during stirring. The mean diameter of bubbles also increased with an increased amount of Tween 20 in the range of concentration between 0.10 and 0.35%, with some fluctuations. Karim and Wai observed that a higher addition of foaming ingredients led to larger bubbles with a broader size distribution [42]. However, the mean size of bubbles was reduced at the highest concentration of Tween 20. Incorporating a high amount of surfactant caused a greater gas fraction to be introduced, which led to the production of smaller bubbles. This can be confirmed by the results of the acoustic tests, where the highest concentration of Tween 20 resulted in the highest number of acoustic events with the highest energy. This may indicate that there were more pores (bubbles) in the material, and during the deformation, the fracture of many pores generated a high acoustic intensity.

**Figure 4:**
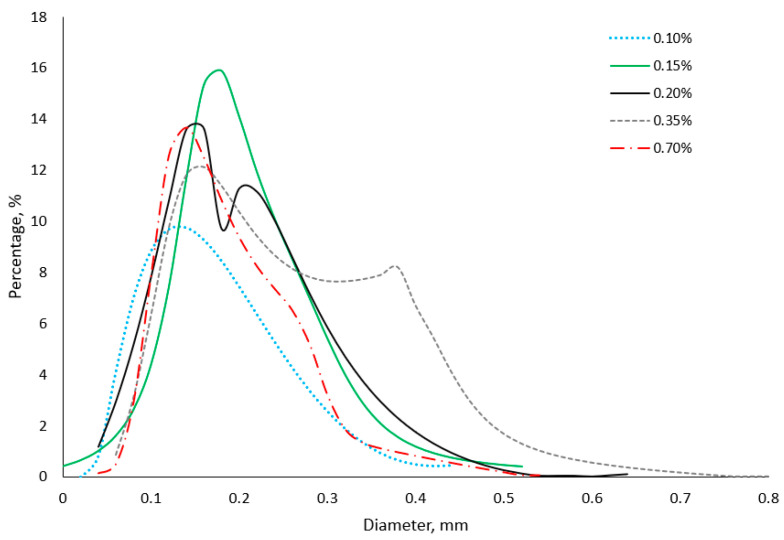
Percentage histogram of pore diameter in agar gels with different additions of Tween 20.

The concentration of the foaming agent affected the aeration level, gas hold-up and dispersion of bubbles in the material [35,43]. Figure 4 presents the diameter size distribution of bubbles in gels. An increase in the addition of Tween 20 from 0.10 to 0.20% caused the distribution of diameter size to shift towards larger values. These gels contained pores with diameters ranging from 0.0001 to 0.64 mm. Bimodal pore size distribution was observed for the gels with Tween 20 concentrations of 0.20 and 0.35%. This kind of behaviour of samples may indicate changes in structure, such as flocculation. This also confirms the results of the TSI, which showed the highest values for this concentration of Tween 20.

**Figure 5:**
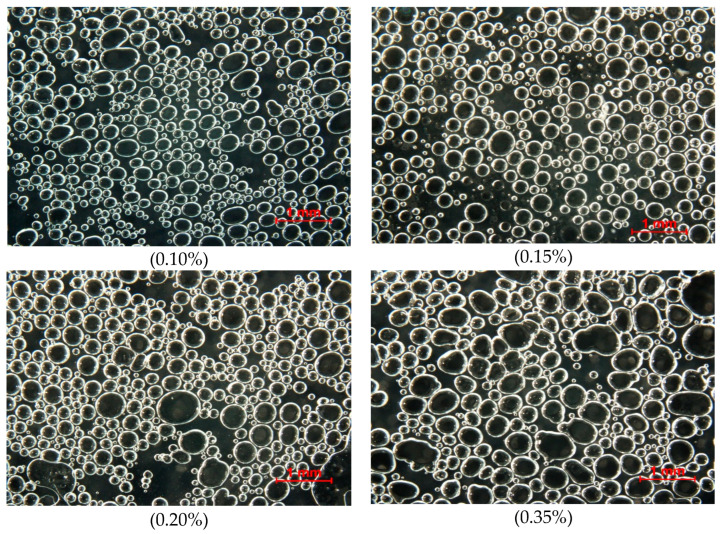

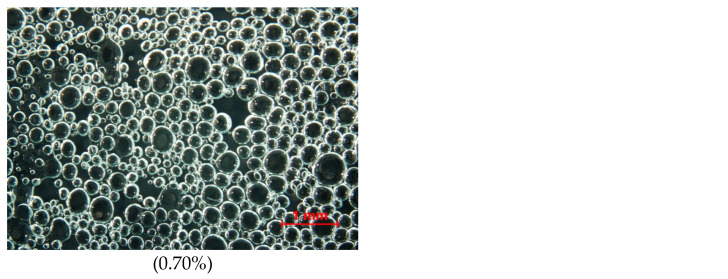
Microscopic photos of agar gels with different concentrations of Tween 20; scale bar: 1 mm.

Images of the structure of the gels show that at a low concentration of Tween 20, small bubbles dominated (Figure 5). An increase in the concentration of the foaming agent led to more heterogenous structures with large and small bubbles. Micro-CT images of aerated 3% agar gel with 0.5% Tween show that initial polydispersed air cells were mainly displayed. The size of bubbles increased, and their number decreased, due to coarsening and disproportionation during storage [33]. An increase in the addition of foaming agents resulted in a more narrow size diameter distribution (range from 0.04 to 0.52 mm) (Figure 4). The microscopic structure also shows that the gel with 0.70% concentration contained tightly packed tiny bubbles (Figure 5). The results of image analysis and the obtained microscopic photos can be related to the mechanical properties of the foamed gels. The introduction of bubbles caused a decrease in hardness and mechanical resistance. However, an increase in the Tween 20 concentration from 0.1 to 0.15% caused the production of a considerably softer gel, which contained a higher number of bigger bubbles. The gel with the addition of Tween 20 at 0.35% showed a slight decrease in compression force, and the material’s structure contained fractions of small and large bubbles. The heterogeneous distribution of bubbles suppressed the integrity of the gel. The presence of many small and more uniform bubbles at 0.7% addition of surfactant led to better integrity of the gel, with similar hardness obtained for gels with a lower concentration of Tween 20.

The results of Pearson’s correlation analysis showed that the mean size of bubbles was positively correlated with the syneresis index and duration of AE events (r = 0.89, r = 0.94; *p* < 0.05), but negatively correlated with the number of AE events (r = –0.84; *p* < 0.05). Springiness was negatively correlated with gas hold-up (r = –0.97; *p* < 0.05). It can be said that the presence of small bubbles led to higher stability of the gel and the generation of more acoustic emission events.

Adding Tween 20 to agar gels can produce a foamed, soft product. The most homogeneous gel structure was obtained at a Tween 20 concentration of 0.70%. The foam structure is essential in the case of desserts. Consumers perceive products such as aerated chocolate or marshmallows as low-calorie products. The structure and texture of foamed products can also be attractive to patients with dysphagia. Gels with the lowest concentration of Tween 20 were characterised by higher hardness than other gels with surfactant. This can be crucial in industrial production, as reduced addition of the same ingredients may decrease fabrication costs. Further studies with sensory analysis may enable us to detect whether consumers better perceive products with higher hardness or porosity.

## 3. Conclusions

Agar gels A and B were characterised by similar density, water activity and gel strength. Introducing Tween 20 to agar gel A led to decreased density and increased gas hold-up of the samples. The addition of Tween 20 in amounts ranging from 0.10 to 0.35% contributed to a gradual decrease in the stability of the gels. Changes in the texture (decrease in TPA attributes) and weakening of the gels were linked with the heterogenous structure of the gels. An increase in the addition of Tween 20 to 0.70% led to a reduced size of bubbles and an increase in their number, better stability and lower gel syneresis. The intensive acoustic emission for this type of gel was related to the presence of many small bubbles. The bubble size distribution at the Tween concentration of 0.10% was wide-ranging, indicating structure heterogeneity. Further increasing the Tween 20 concentration to 0.35% probably caused the bubbles’ rearrangement and coalescence.

It is necessary to determine the optimal concentrations of Tween 20 at which the required material properties are achieved. They may be different depending on the applied hydrocolloid matrix. Further research should determine the extent to which other factors, such as the presence of various ingredients or the environment, may influence the usefulness of Tween 20.

Application of 0.7% Tween 20 enabled the production of foamed gels with a uniform structure and small pores, but low hardness and gumminess. Application of a lower concentration of Tween of 0.1% produced more rigid gels with limited gel syneresis. Adding Tween 20 at the appropriate level can be a factor in obtaining gels with a tailored structure and texture.

## 4. Materials and Methods

### 4.1. Materials

**Figure 6:**
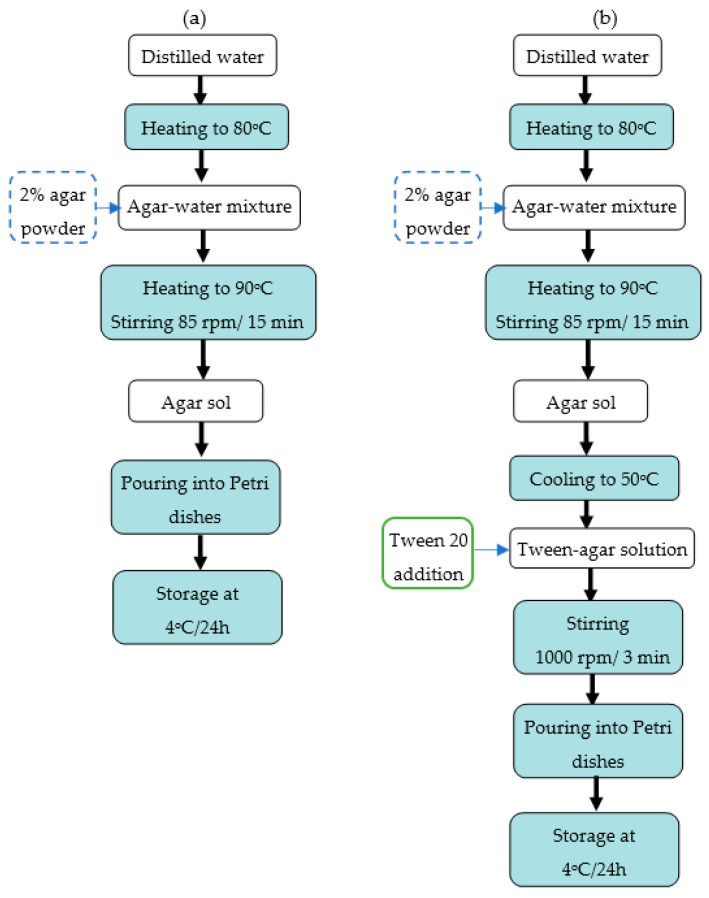
Scheme of preparation of pure agar gel (**a**) and agar gel with Tween 20 addition (**b**).

In the first part of the experiment (Figure 6a), the hydrocolloid gel samples were prepared with the addition of 2% agar powder (MW = 478 g/mol), supplied by two companies: Agnes (Białystok, Poland)—Agar A (MW = 478 g/mol), and Biomus (Lublin, Poland)—Agar B. Water (200 mL) was heated to 80 °C using a hot plate magnetic stirrer (IKA, Staufen, Germany). Then, agar-agar powder A or B was added and mixed homogeneously at 85 rpm. The agar and water mixture was heated to 90 °C for 15 min, until a clear solution was obtained. The agar sol was poured into Petri dishes and stored at 4 °C for 24 h. The selected physical properties of the gelled samples were analysed after storage.

The structured agar gel with Polyoxyethylene sorbitan monolaurate (Tween 20, Sigma-Aldrich, Saint Louis, MI, USA) was prepared in the second part of the experiment (Figure 6b). The preparation procedure of agar sol was analogous to that used for pure agar gel. Polysorbate 20-Tween 20 (MW = 1228 g/mol, Purity GC = 53%) at different concentrations (0.1, 0.15, 0.2, 0.35, 0.7) was added to the agar solution at a temperature of 50 °C, and mixed using a kitchen mixer (Severin, Sundern, Germany) at a speed of 1000 rpm for 3 min. The foamed mixture was poured into Petri dishes. The samples were allowed to cool and solidify. The foamed gels were kept at 4 °C for 24 h in covered Petri glass dishes, and further measurements were performed.

The gelled samples (with or without tween 20) were diced into cubes (12 × 12 × 12 mm) for the performance of mechanical and acoustic tests.

### 4.2. Selected Physical Properties of Agar Gels with and Without Tween 20

#### 4.2.1. Agar A and Agar B Gels

The gels’ water activity (a_w_) was measured using an a_w_ analyser (Aqualab, series 3 Decagon Devices Inc., Pullman, WA, USA) with an accuracy of ±0.001. The density of the gels was calculated based on the measured mass and volume of the samples [11]. The syneresis index was obtained according to the method described by Jakubczyk et al. [41], with some modifications. The gelled samples in falcons were centrifuged for 8 min, at a speed of 3500 rpm, using a 4-15 SIGMA centrifuge with a rotor 12165. The syneresis index was calculated based on the initial gel’s mass (m*_i_*) and the mass of the sample after centrifugation and decantation of the supernatant (m*_k_*).

(1) $$S=\left(\frac{m_{i}-m_{k}}{m_{i}}\right)\cdot 100\%$$

The water activity, density and syneresis measurements were performed with three repetitions at a temperature of 25 °C.

The mechanical resistance of the gels was analysed based on the maximal force recorded during the compression of gel cubes with a constant deformation speed of 1 mm·s^−1^, at a strain of 80%. The tests used a TA-HD plus a texture analyser (Stable Micro Systems, Surrey, UK), with a 5 kg load cell and 23 mm diameter platen. The experiment was performed 20 times for each type of gel.

The rheological properties of the two kinds of agar sols (A and B) were measured using a Haake Mars 40 rheometer (Thermo Scientific Inc., Karlsruhe, Germany). All tests were performed using a parallel plate geometry with a diameter of 35 mm (P35 Ti) and a gap size of 1 mm. Strain sweep tests (frequency 1 Hz, temperature of 25 °C) were conducted to obtain the linear range LVR. Based on the LVR results, the frequency sweep test was conducted at a constant strain of 0.02 and in a frequency range of 0.1–10 Hz (also at a temperature of 25 °C). A temperature sweep test at a rate of 2 °C·min^−1^ was applied during cooling of samples from 50 to 20 °C, with 0.02 strain and at a frequency of 1 Hz. All measurements were triplicated.

#### 4.2.2. Agar A and Agar B Gels with Addition of Tween 20

The density and the mechanical resistance (maximal force) were measured for agar gels A and B with the addition of Tween (0.7–0.7%). The measurement protocol was the same as that applied to pure agar gels.

#### 4.2.3. Characteristic of Agar A Gels with Tween 20

Some physical attributes were measured only for agar A gel with Tween. The water activity and syneresis index were obtained by a method similar to that used for pure gel.

The texture profile analysis (TPA) test was performed in 20 repetitions using a TA-HD plus texture analyser (Stable Micro Systems, Surrey, UK). A double compression test was carried out with a constant deformation speed of 1 mm/s, at a strain of 50%, with a time gap of 5 s. Selected TPA attributes were obtained: hardness (N), springiness, cohesiveness and gumminess (N).

The acoustic properties were measured according to the procedure used by Jakubczyk et al. [11], in 20 replications. During the compression test of gel cubes (the mechanical resistance measurement), an acoustic emission (AE) signal was recorded using a piezoelectric accelerometer type 4381 (Brüel and Kjær Naerum, Denmark). The number of AE events, total AE energy (arbitrary units—a.u.) and duration of AE events (μs) were obtained based on acoustic emission analysis.

The stability of the gel systems was evaluated using Turbiscan Lab^®^ (Formulation SA, Toulouse, France), which collects data at a given frequency regarding the intensity of backscattered light along the height of the sample. The measurement was performed every 24 h for 9 days. The Turbiscan Stability Index (TSI) was determined using the TurbiSoftLab 2.3.1.125 software (Formulation SA, Toulouse, France). The measurements were made in triplicate.

The gas hold-up of gels with Tween (%) was calculated based on the density of the pure gels ρ*_g_* and foamed samples with Tween 20 ρ*_f_* [27].

(2) $$Gas\;hold-up=\left(1-\frac{\rho_{f}}{\rho_{g}}\right)\cdot 100\%$$

The structure of the gels with Tween was analysed using a Nikon SMZ 1500 optical microscope with a camera (Nikon, Tokyo, Japan). Foamed sol was spread on glass microscope slides, and at least seven structure photos were obtained. The structure analysis was performed at 1.5× magnification. The size of air bubbles was determined using the NIS-Elements BR 3.2. software (Nikon Instruments Inc., Melville, NY, USA). This programme obtained histograms showing the pore diameter distribution in the material. Based on the measurement of 300 bubbles, their mean diameter was calculated.

### 4.3. Statistical Analysis

Statistical analysis was performed using Statistica v 13.3 software (StatSoft Inc., Tulsa, OK, USA), based on one-way analysis of variance (ANOVA) and Tukey’s test at the 95% significance level. The results were presented as the mean ± standard deviation. Additionally, Pearson’s correlation analysis was carried out to estimate the relations between the investigated attributes of the gels.

## Figures and Tables

**Table 1:** Selected physical parameters of agar gels A and B.

Parameter	Agar Gel A	Agar Gel B
Water activity	0.985 ± 0.001 ^a^	0.986 ± 0.002 ^a^
Density, g/cm^–3^	1.094 ± 0.001 ^a^	1.095 ± 0.007 ^a^
Syneresis index, %	1.20 ± 0.50 ^a^	1.28 ± 0.40 ^a^
Maximal force, N	24.15 ± 1.80 ^a^	26.14 ± 2.40 ^a^

The same letter (in rows) indicates no differences between the parameters obtained for agar gel A and B.

**Table 2:** TPA parameters of agar gel with different concentrations of Tween 20.

Tween Concentration, %	Hardness, N	Springiness	Cohesiveness	Gumminess, N
0.00	11.71 ± 4.19 ^a^	0.91 ± 0.01 ^a^	0.68 ± 0.02 ^a^	7.68 ± 0.05 ^a^
0.10	1.98 ± 0.19 ^b^	0.89 ± 0.05 ^ab^	0.59 ± 0.03 ^b^	1.16 ± 0.10 ^b^
0.15	0.78 ± 0.11 ^c^	0.83 ± 0.02 ^b^	0.60 ± 0.02 ^b^	0.47 ± 0.07 ^c^
0.20	1.01 ± 0.14 ^c^	0.70 ± 0.04 ^c^	0.59 ± 0.01 ^b^	0.59 ± 0.09 ^c^
0.35	0.75 ± 0.15 ^c^	0.75 ± 0.07 ^bc^	0.56 ± 0.03 ^bc^	0.43 ± 0.10 ^c^
0.70	0.87 ± 0.14 ^c^	0.67 ± 0.04 ^c^	0.54 ± 0.03 ^c^	0.47 ± 0.09 ^c^

The same letter (in columns) indicates no differences between the parameters obtained for the gel.

**Table 3:** Acoustic emission descriptors of agar gel with different concentrations of Tween 20.

Tween Concentration, %	Number of AE Events	Total Acoustic Energy, a.u.	Duration of AE Event, μs
0.00	-	-	-
0.10	110 ± 18 ^a^	22.3 ± 3.2 ^bc^	54 ± 3 ^d^
0.15	79 ± 10 ^b^	17.3 ± 2.8 ^c^	82 ± 4 ^ab^
0.20	56 ± 11 ^c^	25.4 ± 5.0 ^bc^	77 ± 3 ^b^
0.35	62 ± 13 ^bc^	30.2 ± 5.4 ^b^	89 ± 4 ^a^
0.70	120 ± 23 ^a^	60.4 ± 7.4 ^a^	62 ± 2 ^c^

The same letter (in columns) indicates no differences between the parameters obtained for the gel.

**Table 4:** Selected physical parameters of agar gel with different concentrations of Tween 20.

Tween Concentration, %	Gas Hold-Up, %	Syneresis Index, %	TSI	Mean Diameter, mm
0.00	-	3.20 ± 0.50 ^d^	5.06 ± 0.05 ^a^	-
0.10	70.4 ± 0.2 ^d^	5.82 ± 0.47 ^c^	2.35 ± 0.09 ^d^	0.168 ^b^
0.15	73.9 ± 0.2 ^c^	7.12 ± 0.11 ^b^	2.01 ± 0.21 ^e^	0.203 ^a^
0.20	78.7 ± 0.7 ^b^	6.59 ± 0.26 ^bc^	2.75 ± 0.20 ^c^	0.196 ^ab^
0.35	78.0 ± 0.5 ^b^	9.80 ± 0.90 ^a^	4.15 ± 0.11 ^b^	0.251 ^a^
0.70	81.0 ± 0.4 ^a^	5.99 ± 0.14 ^c^	2.05 ± 0.10 ^e^	0.183 ^b^

The same letter (in columns) indicates no differences between the parameters obtained for the gel.

## Data Availability

Data is contained within the article.
